# How do high glycemic load diets influence coronary heart disease?

**DOI:** 10.1186/s12986-015-0001-x

**Published:** 2015-03-08

**Authors:** Marc J Mathews, Leon Liebenberg, Edward H Mathews

**Affiliations:** CRCED, North-West University, and consultants to TEMM International (Pty) Ltd, P.O. Box 11207, Silver Lakes, 0054 South Africa

**Keywords:** High glycemic load, Coronary heart disease, Biomarkers

## Abstract

**Background:**

Diet has a significant relationship with the risk of coronary heart disease (CHD). Traditionally the effect of diet on CHD was measured with the biomarker for low-density lipoprotein (LDL) cholesterol. However, LDL is not the only or even the most important biomarker for CHD risk. A suitably integrated view of the mechanism by which diet influences the detailed CHD pathogenetic pathways is therefore needed in order to better understand CHD risk factors and help with better holistic CHD prevention and treatment decisions.

**Methods:**

A systematic review of the existing literature was conducted. From this an integrated CHD pathogenetic pathway system was constructed. CHD biomarkers, which are found on these pathways, are the only measurable data to link diet with these CHD pathways. They were thus used to simplify the link between diet and the CHD mechanism. Data were systematically analysed from 294 cohort studies of CHD biomarkers constituting 1 187 350 patients.

**Results and discussion:**

The resulting integrated analysis provides insight into the higher-order interactions underlying CHD and high-glycemic load (HGL) diets. A novel “connection graph” illustrates the measurable relationship between HGL diets and the relative risks attributed to the important CHD serological biomarkers.

The “connection graph” vividly shows that HGL diets not only influence the lipid and metabolic biomarkers, but also the inflammation, coagulation and vascular function biomarkers in an important way.

**Conclusion:**

A focus primarily on the low density lipoprotein cholesterol biomarker for CHD risk has led to the traditional guidelines of CHD dietary recommendations. This has however inadvertently led to HGL diets. The influence of HGL diets on the other CHD biomarkers is not always fully appreciated. Thus, new diets or other interventions which address the full integrated CHD impact, as shown in this paper, are required.

## Background

Coronary heart disease (CHD) is the largest cause of death globally [1]. Cholesterol is commonly assumed to be a crucial element of CHD [2]. Therefore dietary recommendations have traditionally focused on the reduction of saturated fatty acids [3]. This has led to the adoption of low-fat, high carbohydrate diets [4]. However, such high-glycemic load (HGL) diets have been shown to increase the relative risk for CHD [3,5].

Forty percent of CHD deaths occur in men and women who have cholesterol levels lower than the average for the general population [6]. The focus on a single biomarker may thus be oversimplified.

But how does a HGL diet influence all the CHD pathogenetic pathways? The authors could not find a study which integrated all the CHD pathways activated by a HGL diet in order to give insight at a glance. This paper thus investigates the interconnectivity of the effects of HGL diets with CHD pathogenetic pathways. We then use CHD biomarkers, which measure the CHD risk of a pathway, to simplify the integrative CHD model.

We can thus investigate all the effects of a HGL diet on CHD risk, as opposed to only the effect of the pathways quantified by one biomarker, namely low-density lipoprotein (LDL) cholesterol.

## Methods

### Search criteria

We searched PubMed, Science Direct, Ebsco Host, and Google Scholar for publications with “coronary heart disease“ or “coronary artery disease” or “cardiovascular disease” or “CHD” as a keyword and combinations with “high glycemic load diets”, “relative risk prediction”, “network analysis”, “pathway analysis”, “interconnections”, “systems biology”, “pathogenesis”, “biomarkers”, “conventional biomarkers”, “drugs”, “therapeutics”, pharmacotherapeutics”, “hypercoagulability”, “hypercholesterolaemia”, “hyperglycaemia”, “hyperinsulinaemia”, “inflammation”, and “hypertension” in the title of the study.

We also searched all major relevant specialty journals in the areas of cardiology, nutrition, endocrinology, psychoneuroendocrinology, systems biology, physiology, CHD, the metabolic syndrome and diabetes, such as *Circulation; Journal of the American College of Cardiology; Arteriosclerosis, Thrombosis and Vascular Biology; The Lancet; New England Journal of Medicine; American Journal of Medicine; Nature Medicine; Diabetes Care; Journal of Clinical Endocrinology and Metabolism; American Journal of Clinical Nutrition; Preventive Medicine; Molecular Psychology;* and *Journal of Physiology* for similar or related articles.

Furthermore, we selected PubMed and Google Scholar for meta-analyses with keywords “coronary heart disease” or “coronary artery disease” or “cardiovascular disease” or “CHD”. We also reviewed articles referenced in primary sources and their relevant citations. However, unless cited more than 50 times, we included only articles published after 1998 as these contained the most relevant data.

### Study selection

Only articles using the following risk measures were included: relative risk (RR), odds ratio (OR), or hazard ratio (HR). It was not the intention of this study to conduct individual meta-analyses of the individual biomarkers or lifestyle effects and thus the most recent meta-analysis of each biomarker was used for the risk data. Where no meta-analysis for CHD risk was available for a specific biomarker or lifestyle effect a single high quality representative study was used.

Only the trends from each meta-analysis that was adjusted for the most confounding variables was used and only where sufficient information was available on that trend. This was done so that the effects of most of the potential confounders could be adjusted for. This may, however, have increased the heterogeneity between studies, as not all studies adjusted for the same confounders.

CHD was classified as the incidence of atherosclerosis, coronary artery disease, or myocardial infarction. Where results were given for cardiovascular disease these were interpreted as CHD only in scenarios where the effect of stroke could be accounted for or results were presented separately. Biomarkers were only considered if they were associated with an increased or decreased risk of CHD.

In a general sense we characterised two different aspects that had an effect on CHD risk from the systems based view of CHD by using RR data. These aspects were the lifestyle effects and the risk associated with increased levels of certain biomarkers. The lifestyle effects were considered as effect versus control. In other words, the RR was calculated for the CHD incidence of a lifestyle versus a control or placebo group. For the biomarkers, however, a different approach had to be used due to the differing levels of markers which are possible *in vivo*.

The RR for HGL diet effects was retrieved from a meta-analysis based on prospective population based studies. The RR data for the biomarkers were also retrieved from meta-analyses based largely on prospective population based studies.

The RR for changes in biomarkers were, where possible, extracted from the most recent meta-analysis conducted on the specific biomarker. If no meta-analysis was available, a suitable high quality study was included. In order to limit errors in comparisons between biomarkers only RR given per increase of 1-standard deviation (SD) in the biomarker level was included. The standardisation of RR to RR per 1-SD prohibits the misrepresentation of risk due to the selection of extreme exposure contrasts [7].

### Data extraction

The following data were extracted from the studies: journal citation; number of cases per lifestyle study for OR, RR and HR; total number of persons, including gender, per study; characterisation and severity of lifestyle; type/intensity of CHD; whether the risk was measured in RR, OR or HR; the risk per lifestyle study, and the 95% confidence intervals per lifestyle study.

### Data analysis

Heterogeneity between studies was inevitable due to the large quantity of meta-analyses considered. Each underlying meta-analysis reported individually on the heterogeneity in their analysis. However, these effects were not so large as to discount the effects observed.

The individual meta-analyses also had detailed accounts of differences between studies and subgroup analyses. However, these aspects are not further elaborated on in this study as they were used as a measure of validity in the study inclusion process. The individual studies selected unfortunately represent only the risk associated with the cohort studied and cannot be accurately extrapolated to other populations without further research.

OR and HR were converted to RR using the approach outlined by Zou [8]. It must however be noted that some of the RR values in this article differ from convention. The need for this comes as a result of the visual scaling of the traditional relative risk. Traditionally, if one plots an RR = 3 and RR = 0.33, respectively, the one does not ‘look’ three times worse and the other three times better than the normal RR = 1. The reason is that the scales for the positive and negative effects are not numerically similar. A graph of ‘good’ and ‘bad’ RR can therefore be deceptive for the untrained person, e.g., a patient.

This article rather uses the method that the conventional RR = 3 is three times worse than the normal RR = 1. While the conventional RR = 0.33 means that the patient’s position is three times better than the normal RR = 1. Thus, in summary: a conventional RR = 3 is presented as per normal, as a 3-fold increase in risk and a conventional RR = 0.33 is presented as a 3-fold decrease in risk (1/0.33 = 3).

## Results

### Integrated model

**Figure 1:**
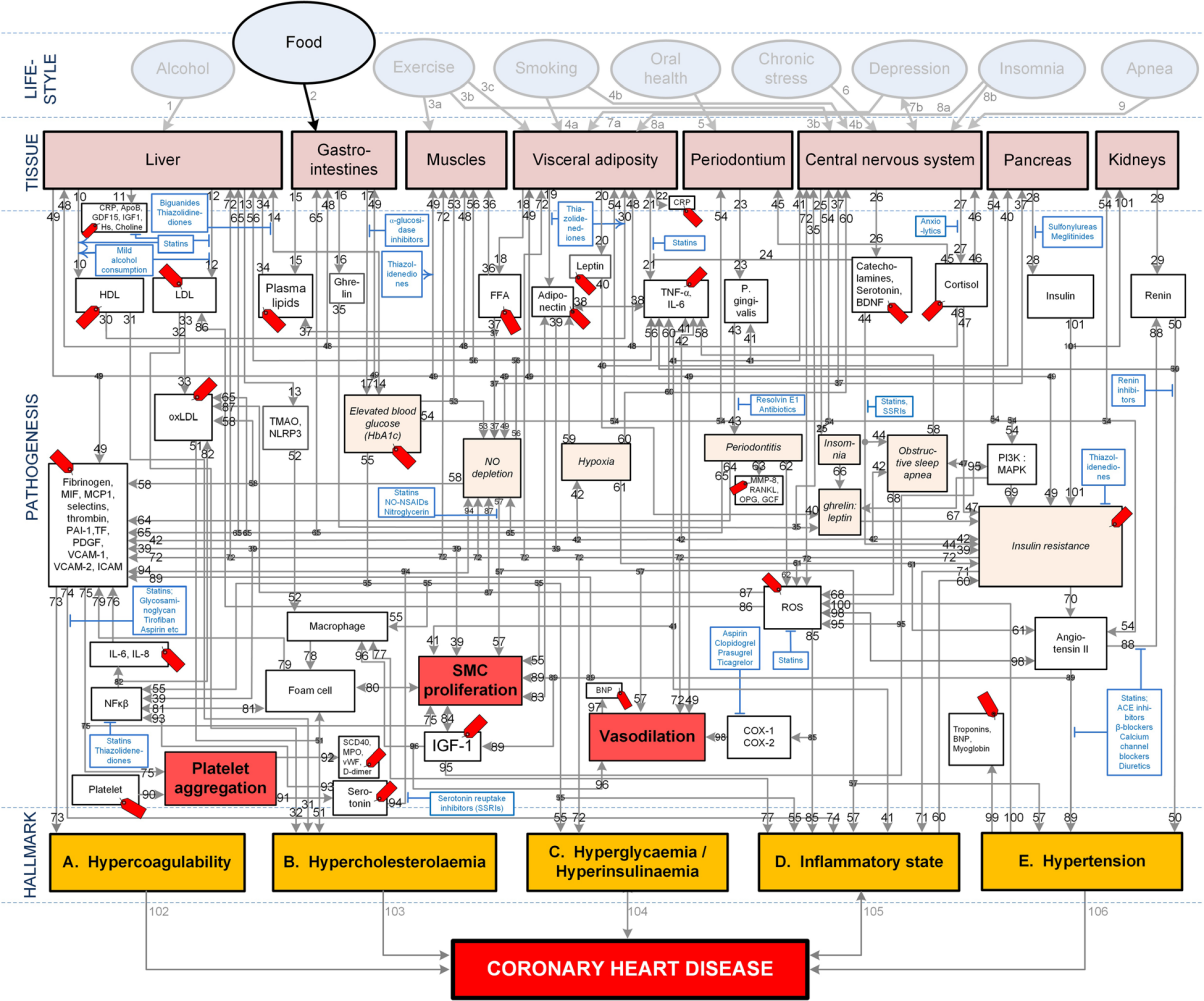
**Conceptual model of general lifestyle effects, salient CHD pathogenetic pathways and CHD hallmarks.** The affective pathway of pharmacotherapeutics, blue boxes, is shown in Figure 1, and salient serological biomarkers are indicated by the  icon. The blunted blue arrows denote antagonise or inhibit and pointed blue arrows denote up-regulate or facilitate. HDL denotes high-density lipoprotein; LDL, low-density lipoprotein; oxLDL, oxidised LDL; FFA, free fatty acids; TMAO, an oxidation product of trimethylamine (TMA); NLRP3, Inflammasome responsible for activation of inflammatory processes as well as epithelial cell regeneration and microflora; Hs, homocysteine; IGF-1, insulin-like growth factor-1; TNF-α , tumour necrosis factor-α; IL, interleukin; NO, nitric oxide; NO-NSAIDs, combinational NO-non-steroidal anti-inflammatory drug; SSRI, serotonin reuptake inhibitors; ROS, reactive oxygen species; NFκβ, nuclear factor-κβ; SMC, smooth muscle cell; HbA_1c_, glycosylated haemoglobin A1c; P. gingivalis, Porphyromonas gingivalis; vWF, von Willebrand factor; PDGF, platelet-derived growth factor; MIF, macrophage migration inhibitory factor; SCD-40, recombinant human sCD40 ligand; MPO, myeloperoxidase; MMP, matrix metalloproteinase; VCAM, vascular cell adhesion molecule; ICAM, intracellular adhesion molecule; CRP, C-reactive protein; PAI, plasminogen activator inhibitor; TF, tissue factor, MCP, monocyte chemoattractant protein; BDNF, brain-derived neurotrophic factor; PI3K, phosphatidylinositol 3-kinase; MAPK, mitogen-activated protein (MAP) kinase; RANKL, receptor activator of nuclear factor kappa-beta ligand; OPG, osteoprotegerin; GCF, gingival crevicular fluid; D-dimer, fibrin degradation product D; BNP, B-type natriuretic peptide; ACE, angiotensin-converting-enzyme; COX, cyclooxygenase; β-blocker, beta-adrenergic antagonists.

The integrated model in Figure 1, which we developed, schematically illustrates the complexity of CHD. (A more detailed discussion of Figure 1 is given in Section “Pathogenetic effects of high glycemic load diets”). It is however important to realize that CHD involves inputs from hundreds of gene expressions and a number of tissues. Thus, analysing the individual components of the system would not be sufficient, as it is important to know how these components interact with each other [9]. For instance, genetic and lifestyle factors influence clinical traits by perturbing molecular networks [10]. A high-level systems-based view of CHD therefore has the potential to interrogate these molecular phenotypes and identify the patterns associated with the disease.

Pathways can be tracked from a chosen lifestyle effect to a hallmark of CHD if the two states are connected by the pathogenesis of the disorder. The pathways are therefore a visual representation of previously published knowledge integrated here.

The pathogenetic pathways of interest for this review were only those between HGL diets (“Food”) and CHD. The effects of other lifestyle effects (e.g. moderate alcohol consumption, moderate intensity exercise, smoking, oral health, chronic stress, depression, insomnia and sleep apnoea) are not considered here.

The lifestyle effect of “Food” (Figure 1) was regarded as HGL diets (daily mean GL ≥ 142). “Tissue” in Figure 1 indicates the organ or type of tissue which is affected by a pathogenetic pathway or trait. “Pathogenesis” in Figure 1 indicates the pathological pathways of the disorder.

Salient serological biomarkers (shown in Figure 1 as ) and pharmacotherapeutics (shown in Figure 1 as ) that act on the pathways are also indicated in Figure 1. These pathogenetic pathways also lead to certain traits (e.g. insulin resistance) that lead to five pathophysiological end-states, which we designate as “hallmarks of CHD”, namely hypercoagulability, hypercholesterolaemia, hyperglycaemia/hyperinsulinaemia, an inflammatory state, and hypertension.

The formulation of this conceptual model required the consultation of numerous publications. The journal references which were used to describe the main pathogenetic pathways in the model are given in Table 1. It is however not the purpose of this review to describe in detail all these pathways. The aim is merely to simplify Figure 1 to show only the pathways relevant to HGL diets.

**Table 1:** **Pathogenetic pathways (in Figure** **1** **) and cited works**

**Pathway**	**Refs.**	**Pathway**	**Refs.**	**Pathway**	**Refs.**	**Pathway**	**Refs.**	**Pathway**	**Refs.**	**Pathway**	**Refs.**
**1**	[11,12]	**2**	[13-17]	**3 a,b,c**	[18-20]	**4 a,b**	[21-23]	**5**	[24-26]	**6**	[27-29]
**7 a,b**	[30-35]	**8 a,b**	[36-38]	**9**	[39]	**10**	[40,41-44]	**11**	[44,45]	**12**	[44]
**13**	[15-17]	**14**	[45-53]	**15**	[52-54]	**16**	[36-38]	**17**	[46-53]	**18**	[23,55-57]
**19**	[54,55]	**20**	[36-38]	**21**	[45,57-63]	**22**	[57]	**23**	[64-68]	**24**	[69-71]
**25**	[36-38]	**26**	[69-74]	**27**	[28,29,75-87]	**28**	[88-92]	**29**	[44,93]	**30**	[40,41-45]
**31**	[40,41-45]	**32**	[44]	**33**	[44]	**34**	[45,54-57]	**35**	[36-38]	**36**	[45,54-57]
**37**	[45,54-57]	**38**	[61,86,94-98]	**39**	[54,55]	**40**	[36-38]	**41**	[60,61,98]	**42**	[60,92]
**43**	[24,60,61,64-68]	**44**	[69-71]	**45**	[27,79,81]	**46**	[27,79,81]	**47**	[27,79,81]	**48**	[27,79,81]
**49**	[88-90,99]	**50**	[44,95,100]	**51**	[9,10,44,45,93,94,100-104]	**52**	[15,16]	**53**	[45-53]	**54**	[45-53]
**55**	[45-53,105-110]	**56**	[45,54-57]	**57**	[45,54-57,93,111-114]	**58**	[45,54-57,93]	**59**	[82-85]	**60**	[82-85]
**61**	[82-85]	**62**	[24,65]	**63**	[64-67]	**64**	[24,25]	**65**	[24,25,66]	**66**	[36-38]
**67**	[36-38]	**68**	[54-57]	**69**	[88]	**70**	[88-90]	**71**	[44,88-90,93,115,116]	**72**	[44,88-90,93,115,116]
**73**	[40,45,92]	**74**	[40,45,92]	**75**	[40,63,92,105,112]	**76**	[45,60,61]	**77**	[60,105]	**78**	[60,105]
**79**	[40,45,60,105]	**80**	[40,45,60,105]	**81**	[40,60,105]	**82**	[40,88,94]	**83**	[107-110]	**84**	[60]
**85**	[45,94,101,113,114]	**86**	[45,94]	**87**	[94]	**88**	[44,94,112,115,116]	**89**	[44,94,112,115,116]	**90**	[40,105,112]
**91**	[69-71]	**92**	[40,44,102]	**93**	[69,70]	**94**	[117-120]	**95**	[121-124]	**96**	[121-124]
**97**	[44]	**98**	[40,60,94,105]	**99**	[44]	**100**	[94]	**101**	[88-90]	**102**	[44,46,49,88,94,102]
**103**	[44,45,60,62]	**104**	[44,45,94,102,112]	**105**	[44,45,60,62,125,126]	**106**	[44,45,94,102,112]				

a,b,c denotes multiple pathways between lifestyle effects and CHD pathogenesis.

Despite the rich body of existing knowledge pertaining to CHD pathogenesis, lifestyle effects, and pharmacotherapeutics [9,10,45,101], a suitably integrated high-level conceptual model of CHD could not be found. A high-level model that consolidates the effects of HGL diets on relative risk of CHD and CHD biomarkers was therefore developed. This model could thus help elucidate the higher-order interactions underlying CHD [9] and provide new insights into dietary interventions.

### Pathogenetic effects of high-glycemic load diets

Figure 1 indicates all possible pathogenetic pathways between the various lifestyle effects and CHD. In the present review only the CHD effects of HGL diets are appraised. The pathogenetic pathways which are activated by HGL diets are elucidated in Table 2. It is important to note that not all the pathogenetic pathways indicated in Figure 1 will be relevant in all patients, and all the pathways may not be active simultaneously.

**Table 2:** **Putative effects of high glycemic load diets and salient CHD pathogenetic pathways**

***Lifestyle***	***Pathways, and pathway numbers corresponding to those in Figure*** **2**	***Refs.***
**High- GL diets**	a. 2-↑17-14-↑ blood glucose-55-↑ hyperglycaemia	a. [13,57,103]
	b. 2-↑17-14-↑ blood glucose-54-19-↓ adiponectin-38-↑ TNFα-56-12-↑ LDL-33-↑ oxLDL-51-↑ hypercholesterolaemia	b. [13,57,103]
	c. 2-↑17-14-↑blood glucose-54-↑ PI3K:MAPK-69-↑ insulin resistance-70-↑ angiotensin II-89-↑ hypertension-100-↑ROS-85-↑ inflammatory state	c. [13,57,95,105]
	d. 2-↑17-14-↑ blood glucose-54-↑ PI3K:MAPK-69-↑ insulin resistance-70-↑ angiotensin II-88-50-↑ TNFα-41-↑ inflammatory state	d. [101]
	e. 2-↑17-14-↑ blood glucose-54-↑ PI3K:MAPK-69-↑ insulin resistance-70-↑ angiotensin II-89-↑ SMC proliferation	e. [62]
	f. 2-↑17-14-↑ blood glucose-54-↑ PI3K:MAPK-69-↑ insulin resistance-70-↑ angiotensin II-89-↓ IGF1-84-↑ SMC proliferation	f. [121-123]
	g. 2-↑17-14-↑ blood glucose-54-↑ PI3K:MAPK-69-↑ insulin resistance-70-↑ angiotensin II-89-↑ VCAM1/MCP1-73-↑ hypercoagulability	g. [60]
	h. 2-↑17-14-↑ blood glucose-54-↑ PI3K:MAPK-69-↑ insulin resistance-72-↑ platelet factors-73-↑ hypercoagulability	h. [40,48]
	i. 2-↑17-14-↑ blood glucose-54-19-↓ adiponectin-38-↑ TNFα-41-↑ P. gingivalis-43-↑ periodontitis-64-↑ platelet factors-73-↑ hypercoagulability	i. [27,40,48,66]
	j. 2-↑17-14-↑ blood glucose-54-19-↓ adiponectin-39-↑ insulin resistance	j. [101]
	k. 2-↑17-14-↑ blood glucose-54-19-↓ adiponectin-39-↑ SMC proliferation	k. [94]
	l. 2-↑17-14-↑ blood glucose-54-↑ PI3K:MAPK-69-↑ insulin resistance-72-↑ hyperglycaemia	l. [88,89]
	m. 2-↑17-14-↑ blood glucose-55-↑ SMC proliferation	m. [103]
	n. 2-↑17-14-↑ blood glucose-53-↑ NO depletion-57-↑ SMC proliferation	n. [28,89,105,111]
	o. 2-↑17-14-↑ blood glucose-53-↑ NO depletion-57-↓ vasodilation	o. [28,97,103,105]
	p. 2-↑17-14-↑ blood glucose-54-60-↑insulin resistance-72-↓ vasodilation	p. [101,103]
	q. 2-↑17-14-↑ blood glucose-54-↑angiotensin II-89-↑hypertension-100-↑ROS-85-↑inflammatory state	q. [28,93,103]
	r. 2-↑15-34-12-↑ LDL-33-↑ oxLDL-51- ↑ hypercholesterolaemia	r. [14]
	s. 2-↑15-34-13-↑ TMAO/NLRP3-52-macrophage-78-foam cell-↑ SMC proliferation	s. [15-17]
	t. 2-↑15-34-13-↑ TMAO/NLRP3-52-macrophage-51-↑ hypercholesterolaemia	t. [15-17]
	u. 2-↑15-34-13-↑ TMAO/NLRP3-52-macrophage-77-↑ inflammatory state	u. [15-17]

↑denotes upregulation/increase, ↓denotes downregulation/decrease, *x-y-z* indicates pathway connecting *x* to *y* to *z*. HDL, high-density lipoprotein; LDL, low-density lipoprotein; oxLDL, oxidised LDL; FFA, free fatty acids; TNFα, tumour necrosis factor-α; IL6, interleukin-6; NO, nitric oxide; ROS, reactive oxygen species; BDNF, brain-derived neurotrophic factor; OSA, obstructive sleep apnoea; SMC, smooth muscle cell; P. gingivalis, Porphyromonas gingivalis; PI3K, phosphatidylinositol 3-kinase; MAPK, mitogen-activated protein (MAP) kinase; PI3K:MAPK, ratio of PI3K to MAPK; IGF 1, insulin-like growth factor-1; VCAM 1, vascular cell adhesion molecule-1; MCP 1, monocyte chemoattractant protein-1.

Figure 1, *Pathway: 2-17-14-blood glucose-55-hyperglycaemia* shows how HGL diets are connected to hyperglycaemia through the increase of blood glucose due to carbohydrate consumption [127]. The resulting state of hyperglycaemia and concomitant hyperinsulinaemia are both CHD hallmarks in non-diabetic patients [128]. (Figure 1, Pathway: 2-17-14-blood glucose-55-hyperglycaemia).

The hyperglycaemia that result from HGL diets can also lead to an increase in the PI3K-to-MAPK ratio, through inhibition of the phosphatidylinositol 3-kinase (PI3K) insulin signalling pathway or the stimulation of the MAPK pathway [89]. This in turn increases insulin resistance [129]. (Figure 1, Pathway: 2-17-14-blood glucose-54-PI3K:MAPK-69-insulin resistance).

Decreased insulin sensitivity, due to insulin resistance, has been associated with increases in the serum levels of platelet factors, such as fibrinogen [130] and von Willebrand factor [131], and thus increased potential for hypercoagulability which is a CHD hallmark [132,133]. (Figure 1, Pathway: 2-17-14- blood glucose-54-PI3K:MAPK-69-insulin resistance-72-platelet factors-73-hypercoagulability).

Further, decreased adiponectin levels can result from increased adipose tissue levels stemming from excessive dietary intake due to HGL diets [86]. Decreases in plasma adiponectin concentrations can also decrease insulin sensitivity by decreasing muscle fat oxidation [134] and subsequently cause increased vasodilation [86] which is a hallmark of CHD. Additionally, it is possible for decreases in adiponectin levels to increase those of intramyocelular triacylglycerol which are correlated to insulin resistance [135,136]. (Figure 1, Pathway: 2-17-blood glucose-54-19-adiponectin-39-insulin resistance-vasodilation).

Figure 1 also shows why an insulin-resistant state may be pro-inflammatory, with the expression of the inflammatory mediator TNF-α by adipose tissue being a core aspect associated with plasma insulin [134]. Additionally, adipose tissue has been shown to express other pro-inflammatory mediators, including C-reactive protein (CRP). Macrophages residing in the adipose tissue may also be a source of pro-inflammatory factors by modulating the secretory activities of adipocytes [137]. (Figure 1, Pathway: 2-15-34-13-TMAO/NLRP3-52-macrophage-77-inflammatory state).

HGL diets can also lead to the accumulation of visceral fat, reduced lipoprotein lipase activity and reduced clearance of triglycerides. This leads to increased LDL levels, decreased high-density lipoprotein (HDL) levels, and increased LDL-to-HDL ratios [138], and eventually to hypercholesterolaemia [128] which contributes significantly to atherogenecity, leading to CHD [139]. (Figure 1, Pathway: 2-15-34-12-LDL-33-oxLDL-51-hypercholesterolaemia).

The CHD hallmark hypertension is directly correlated with visceral fat mass [140]. Hypertension may also be mediated through increased vascular and sympathetic tone created by reduced bioavailability of nitric oxide (NO) because of oxidative stress, and increased expression of angiotensinogen by adipose tissue leading to an activation of the renin-angiotensin system [141,142]. (Figure 1, Pathway: 2-17-14-blood glucose-54-angiotensin II-89-hypertension).

From the above high-level model, it is apparent that HGL diets have multiple effects on the pathogenetic mechanism of CHD. Therefore, it can be seen that with greater activation of the pathways connected to the hallmarks of CHD, a patient’s risk of CHD is further amplified. Thus, an integrated multi-faceted approach to therapeutics and lifestyle factors is necessary.

### Biomarkers of coronary heart disease

The integrated model that was developed is a high-level conceptual model, from which the interconnectedness of CHD is immediately apparent (Figure 1). The model is however complicated, thus a novel approach was used to simplify it with regards to the consumption of a HGL diet. In order to simplify the integrated model, serological biomarkers (which can be easily measured) were used to link the effect of HGL diet to the corresponding CHD pathways.

Biomarkers are used as indicators of an underlying disorder or pathogenetic pathway, such as systemic inflammation that is a known aggravating factor in the pathogenesis of CHD [60,61,143]. The measurement of specific biomarkers therefore enables the prediction of the relative risk for CHD associated with these biomarkers [44]. As it is possible to accurately measure certain serum biomarker levels, they can also be used as patient-specific links to pathogenetic, lifestyle (e.g. diet) or pharmacotherapeutic (e.g. α-glucosidase inhibitors) factors. In essence, the biomarkers can be used to indicate the activation of underlying pathogenetic pathways of the disorder. The biomarkers associated with different pathways are indicated in Figure 1 as .

Important CHD biomarkers which have been noted to change with chronic consumption of HGL diets are hyperglycaemia as represented by changes in the glycated haemoglobin levels [144] and hyperinsulinaemia as represented by increased serum insulin levels [145]. Further, additional biomarkers of interest would be the traditional cholesterol levels of LDL and HDL, which have both been noted to be affected by excessive consumption of HGL diets [145].

**Figure 2:**
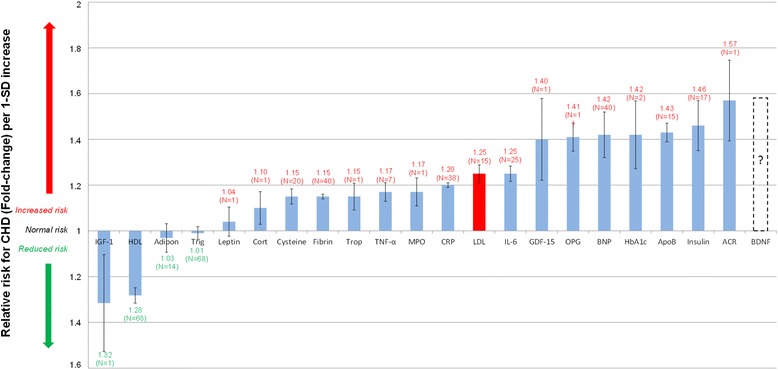
**Normalised relative risks (fold-change) of salient current and potential biomarkers for CHD.** Increased IGF-1 and HDL levels are associated with a moderately decreased CHD risk. (IGF-1 and HDL levels are significantly inversely correlated to relative risk for CHD.) N indicates number of trials; I, standard error; Adipo, adiponectin; HDL, high-density lipoprotein; BNP, B-type natriuretic peptide; ACR, albumin-to-creatinine ratio; GDF-15, growth-differentiation factor-15; Cysteine, Homocysteine; LDL, low-density lipoprotein; HbA_1c_, glycosylated haemoglobin A1c; Trop, troponins; Trigl, triglycerides; CRP, C-reactive protein; IL-6, interleukin-6; Fibrin, fibrinogen; Cort, cortisol; TNF-α, tumour necrosis factor-α; ApoB, apolipoprotein-B; IGF-1, insulin-like growth factor-1; MPO, myeloperoxidase; RANKL or OPG, osteoprotegerin; BDNF, brain-derived neurotrophic factor.

The authors could however not find a published study where all the important serum biomarkers were compared in order to show their relative importance regarding CHD risk prediction in terms of relative risk. We therefore attempted this in Table 3 and the results thereof are presented graphically in Figure 2.

**Table 3:** **Salient serological and functional biomarkers of CHD, and prospective ones**

**Biomarker (class and salient examples)**	**Prediction of CHD relative risk (95% CI)**	**Size of studies** ***(N*** **= number of trials,** ***n*** **= number of patients)**	**Ref.**
*Lipid-related markers:*			
Triglycerides	0.99 (0.94-1.05)	(*N* = 68, *n* = 302 430)	[146]
LDL	1.25 (1.18-1.33)	(*N* = 15, *n* = 233 455)	[147]
HDL	0.78 (0.74-0.82)	(*N* = 68, *n* = 302 430)	[146]
ApoB	1.43 (1.35-1.51)	(*N* = 15, *n* = 233 455)	[147]
Leptin	1.04 (0.92-1.17)	(*n* = 1 832)	[148]
*Inflammation markers:*			
hsCRP	1.20 (1.18-1.22)	(*N* = 38, *n* = 166 596)	[149]
IL-6	1.25 (1.19-1.32)	(*N* = 25, *n* = 42 123)	[150]
TNF-α	1.17 (1.09-1.25)	(*N* = 7, *n* = 6 107)	[150]
GDF-15	1.40 (1.10-1.80)	(*n* = 1 740)	[151]
OPG	1.41 (1.33-1.57)	(*n* = 5 863)	[152]
*Marker of oxidative stress:*			
MPO	1.17 (1.06-1.30)	(*n* = 2 861)	[153]
*Marker of vascular function and neurohormonal activity:*			
BNP	1.42 (1.24-1.63)	(*N* = 40*, n* = 87 474)	[154]
Homocysteine	1.15 (1.09-1.22)	(*N* = 20, *n* = 22 652)	[155,156]
*Coagulation marker:*			
Fibrinogen	1.15 (1.13-1.17)	(*N* = 40*, n* = 185 892)	[149]
*Necrosis marker:*			
Troponins	1.15 (1.04-1.27)	(*n* = 3 265)	[157]
*Renal function marker:*			
Urinary ACR	1.57 (1.26-1.95)	(*n* = 626)	[158]
*Metabolic markers:*			
HbA_1c_	1.42 (1.16-1.74)	(*N* = 2, *n* = 2 442)	[159]
IGF-1	0.76 (0.56-1.04)	(*n* = 3 967)	[160]
Adiponectin	0.97 (0.86-1.09)	(*N* = 14, *n* =21 272)	[161]
Cortisol	1.10 (0.97-1.25)	(*n* = 2 512)	[162,163]
BDNF	**?**	N/A	[71,73,74]
Insulin resistance (HOMA)	1.46 (1.26-1.69)	(*N* = 17, *n* = 51 161)	[164]

Only recent and/or highly cited papers have been cited here. *n* denotes number of participants; *N*, number of trials; ?, a possible, though not currently quantified effect on CHD risk; HDL, high-density lipoprotein; BNP, B-type natriuretic peptide; ACR, albumin–to-creatinine ratio; GDF-15, growth-differentiation factor-15; LDL, low-density lipoprotein; HbA_1c_, glycosylated haemoglobin A_1c_; hsCRP, high-sensitivity C-reactive protein; IL-6, interleukin-6; TNF-α, tumour necrosis factor-α; ApoB, apolipoprotein-B; IGF-1, insulin-like growth factor-1; MPO, myeloperoxidase; RANKL or OPG, osteoprotegerin; BDNF, brain-derived neurotrophic factor; HOMA, homeostatic model assessment.

Table 3 presents the relative risk data from 294 cohort studies comprising 1 161 560 subjects. The results from the studies were thus interpreted and the averaged relative risks (with standard error (I) and study size (N)) were used to populate Figure 2. Figure 2 visually compares the RR associated with serological biomarkers per 1-standard deviation increase in said biomarker.

The main outcome from the relative risk comparison in Figure 2 is that it allows one to compare the relative risk of CHD associated with changes in certain biomarkers. From the figure, it is clear that adverse changes in certain biomarkers, such as ApoB, present a much greater risk than the generally considered LDL cholesterol (Shown in Figure 2 in red). Additionally, glycated haemoglobin A_1c_ (HbA_1c_), an easy-to-measure biomarker that is well correlated with HGL diets [144], is associated with a large increased risk. This type of consideration thus alludes to biomarkers such as ApoB and insulin resistance that are potentially more important for lifestyle and pharmaceutical interventions.

Although the numerical values of relative risk presented in this study are based on large, clustered clinical trials, and thus give a good idea of *average* effects, it is acknowledged that individual patients will have very specific CHD profiles. However, Figure 1 is still relevant to everyone and should thus provide general insight into relevant risk factors. Therefore, Figure 1 could *inter alia* reveal further pathways still available for biomarker and drug discovery.

### Effects of high-glycemic load diets

The pathogenesis of different lifestyle effects are illustrated in Figure 1 and the specific paths regulated by HGL diets are detailed in Table 2. It is therefore possible to quantify the effects of HGL diets on the RR of CHD using Figure 1 as a model for the pathogenesis of CHD. By considering the pathogenesis of HGL diets, the pathways activated thereby are elucidated in Figure 1. Certain pathways might be quantified by the measurement of specific biomarkers (shown as  in Figure 1).

**Figure 3:**
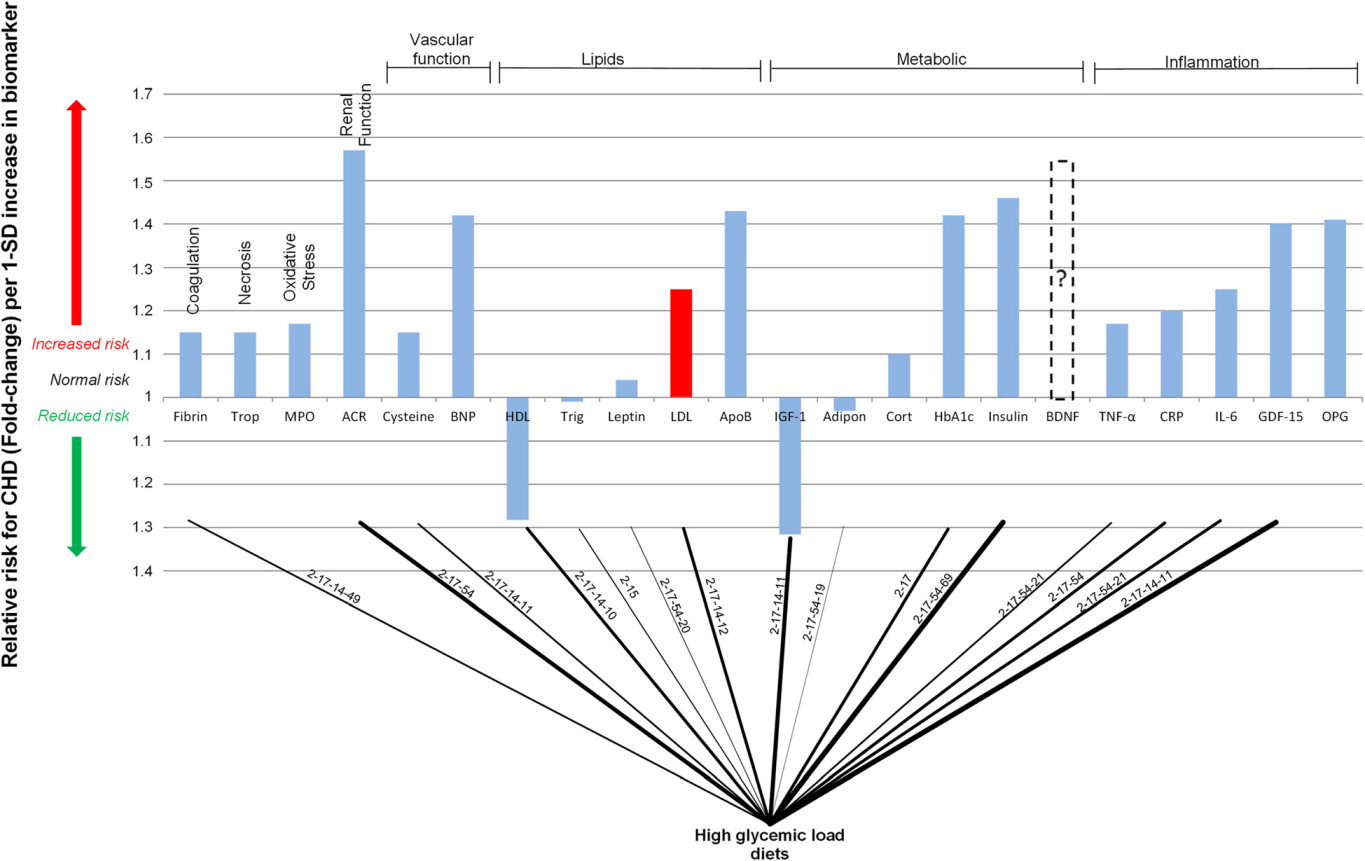
**Interconnection of relative risk effects of high glycemic load diets and serological biomarkers for CHD.** “ACR” denotes albumin-to-creatinine ratio; Trop, troponins; Fibrin, fibrinogen; MPO, myeloperoxidase; BNP, B-type natriuretic peptide; Cysteine, Homocysteine; HDL, high-density lipoprotein; LDL, low-density lipoprotein; Trigl, triglycerides; ApoB, Apolipoprotein-B; Adipon, adiponectin; HbA_1c_, glycosylated haemoglobin A_1c_; Cort, cortisol; IGF-1, insulin-like growth factor-1; BDNF, brain-derived neurotrophic factor; GDF-15, growth-differentiation factor-15; CRP, C-reactive protein; IL-6, interleukin-6; TNF-α, tumour necrosis factor-α; RANKL or OPG, osteoprotegerin.

The effects of HGL diets on CHD are further characterised by the ‘connection graph’ in Figure 3. The ‘connection graph’ is a simplification of the pathogenesis of CHD presented in Figure 1. Within this graph none of the underlying pathogenesis is neglected, but only the CHD biomarkers affected by HGL diets are indicated. The pathways, from Figure 1, through which the consumption of HGL diets effect the biomarkers are shown on the connections.

To make further deductions from the ‘connection graph’ the biomarkers have been sorted into classes in terms of their clinical effect. The classes are renal function, necrosis, coagulation, oxidative stress, vascular function, lipids, metabolic function and inflammation. The ‘connection graph’ therefore allows easy visual recognition of the effects of different lifestyle factors, in this case HGL diets on the biomarkers of CHD.

The pathogenetic pathways (from Figure 1) are superimposed on the connecting lines in Figure 3. Therefore, increasing line thickness indicates a connection with greater pathogenetic effect (as quantified by the biomarker’s relative risk prediction of CHD). For example, the relative risk of CHD is relatively low when considering leptin, thus the connection line between HGL diets and leptin is thin.

From the connection graph, it is clear that there are many connections between HGL diets and the biomarkers of CHD. Firstly, it is rather evident that chronic consumption of a HGL diet would serve to induce chronic hyperglycaemia [165]. This chronic hyperglycaemia will be evident in increased HbA_1c_ levels [166] which predicts an increased RR of CHD [159].

Since hyperglycaemia stimulates insulin secretion [167], chronic hyperglycaemia could also serve to increase insulin resistance, by the over-production of insulin [131]. Insulin resistance, which predicts an increased RR of CHD [164], is associated with hyperinsulinaemia [168].

The metabolic marker adiponectin (Figure 3) is also linked to HGL diets, through increased obesity and visceral adiposity possible from HGL diets [169] which are known to reduce the plasma levels of adiponectin [170].

Increased fibrinogen levels, a coagulation biomarker in Figure 3, are postulated to be caused by increased insulin resistance [130], however this pathogenesis is not fully understood. It is however clear that there is some causal relationship between increased serum insulin levels and increased fibrinogen levels [130,131,171] and a possible state of hypercoagulation. Therefore HGL diet induced insulin resistance may have an effect on coagulation, which is a hallmark of CHD.

It has been found that high carbohydrate diets can affect changes in lipid profile, regardless of the cholesterol, protein or fat content [172,173]. Similar trends are observed in HGL diets which have been found to provide reductions in HDL levels and increased LDL and triacylglycerol levels [55,174] as shown in Figure 3. These results suggest that HGL diets have an attributable effect on the traditional CHD biomarkers HDL and LDL.

Therefore, it can be seen that HGL diets affect all of the aforementioned serological biomarkers in such a manner that the risk for CHD would be increased. The negative effects of HGL diets on a patient’s risk for CHD can thus be quantified in a general sense through the consideration of the connection graph in Figure 3. Furthermore, it is possible to consider patient-specific reactions to HGL diets by measuring said patients biomarker levels.

It is thus evident that two of the major aspects of HGL diets which serve to increase the relative risk for CHD would be the hyperglycaemia and hyperinsulinaemia that may result from these diets. Both these factors are also associated with a greatly increased risk for CHD.

Further potential mediation of CHD risk may also be due to increased fibrinogen levels as a result of hyperinsulinaemia. HGL diets also have adverse impacts on lipids levels through decreased levels of HDL and increased levels of LDL, both conditions of which serve to increase the risk of CHD.

In general, based on a recent meta-analysis of eight studies where modest heterogeneity was present [175], HGL diets are associated with an increased RR of 1.36 (95% confidence interval 1.13 to 1.63). This smaller-than-expected RR effect can be somewhat explained by the heterogeneity of the study, i.e. the difference in risk between men and women. In general, women have been found to have a higher relative risk for CHD in association with HGL diets [3,175].

Heterogeneity is to be expected in the combined risk for CHD as some studies have found that there is no increased risk due to HGL diets in men [176], while other studies have found no increased risk association with women [177].

## Discussion

As can be seen from the preceding discussion, the adoption of HGL diets can have negative impacts on the pathogenesis of CHD which is evident through the modification of several CHD biomarkers. The implication from this is that an increased risk for CHD is observed with the consumption of HGL diets. It is therefore the opinion of the authors that modern dietary guidelines for patients at risk of CHD should reflect this as there is an inadvertent danger of consuming a HGL diet based on current dietary guidelines.

The latest AHA dietary guidelines have attempted to focus on overall diet quality, rather than on specific macronutrient content. Some emphasis was placed on restricting or increasing the consumption of certain types of foods, such as increasing high-fibre foods and decreasing high-trans-fat foods [4]. However, these and previous guidelines have inadvertently caused the adoption of high-carbohydrate diets in order to increase fibre intake and reduce trans-fats [173,178,179] which may lead to HGL diets.

It is acknowledged that the intent of the AHA guidelines was never to increase carbohydrate intake, but instead to increase the intake of fibre through high-fibre carbohydrates and to decrease the consumption of saturated fats. Unfortunately, many patients opt for foods that do not meet the required fibre consumption guidelines [178] which results in the inherent carbohydrates imparting a greater GL, which has been negatively associated with CHD risk in this paper and others [180].

Much of the problems with the dietary recommendations as described by the AHA is the eventual use of high-carbohydrate content foods. It has been proven that high- carbohydrate diets have adverse effects on many of the risk factors which are targeted by the AHA guidelines, including lipid profiles and blood glucose levels [172,178]. A comparison of three different diets by McAuley and co-workers showed that the use of the traditional AHA guideline diet proved to be the worst of the three diets for mediating the risk factors for CHD [178].

Dietary recommendations have long been focused on the type of ingested food [4,181]. However, it has recently become more evident that the type of food ingested is less important than the overall amount of calories ingested [173,178]. Therefore, adherence to any low calorie diet is more important than the specific type of diet [182].

Thus an easy-to-follow and understand diet is obviously required in order to adequately address the issue of “heart healthy” diets and CHD. It is clear from Figure 1 that there is an abundance of links between the hallmarks of CHD and hyperglycaemia and insulin resistance from HGL diets. This was highlighted in the discussion of the pathways that are activated by HGL diets.

The importance of hyperglycaemia and insulin resistance is further highlighted by the increased risks associated with each prospective biomarker [159,164]. As the effects of HGL diets are largely dependent on carbohydrate absorption into the blood stream [127], it may be interesting to consider the effect of inhibiting this absorption. In the integrated system, in Figure 1, the pathway representing carbohydrate absorption is pathway-17, which as indicated can be regulated with the use of α-glucosidase inhibitors [183].

The α-glucosidase inhibitors thus give some insight into the effect of reduced carbohydrate consumption, as would be possible to achieve with a low GL diet. The α-glucosidase inhibitor acarbose has been successfully employed to counteract the effects of carbohydrates in diabetic patients [184,185].

The use of α-glucosidase inhibitors serves to delay the breakdown of carbohydrates in the gut, which slows down the absorption of sugars [183]. This reduces plasma glucose levels, which in turn reduces the requirement of plasma insulin, both risk factors for CHD (Figure 2).

If one then considers that the use of acarbose in diabetic patients resulted in a much lower incidence of CHD according to a meta-analysis of seven studies compromising 2180 patients. It was found that the RR for CHD was 0.36 (95% CI 0.16 to 0.80) in diabetic patients using acarbose compared to the control group [186]. This equates to a 2.78-fold reduction in CHD risk when using our notation.

This substantial relative risk reduction achieved with acarbose [186] accentuates the importance of the specific path on which this pharmacotherapeutic acts (Pathway 17). Through the inhibition of carbohydrate digestion in the stomach, α-glucosidase inhibitors reduce blood glucose levels (HbA_1c_) and reduce insulin levels, increasing insulin sensitivity. Therefore, if α-glucosidase inhibitors are effective to regulate blood glucose levels and insulin resistance, then much of the risk reduction can be explained by the combined effects of decreased blood glucose levels and increased insulin sensitivity [187].

It is important to note that the CHD risk reduction effects that have been observed from treatment with α-glucosidase inhibitors were found in studies on patients with type 2 diabetes mellitus [186]. It is thus conceivable that the reductions in CHD risk achieved could be greater than expected due to the increased risk for CHD associated with type 2 diabetes mellitus [188]. However the underlying effect of α-glucosidase inhibitors on blood glucose and insulin levels may retain it as a suitable candidate for treatment and prevention of CHD in non-diabetic patients.

The effectiveness of α-glucosidase inhibitors in reducing CHD risk in diabetic patients clearly elucidates the importance of the main pathways which they regulate with regards to CHD. This may therefore indicate the importance of regulating these pathways in non-diabetic patients to prevent CHD, such as through the adoption of low GL diets.

## Conclusions

The authors were intrigued by the possible negative effects of HGL diets on a patient’s risk for CHD as well as the over emphasis of LDL cholesterol. As LDL is not the only or even the most important biomarker for CHD risk, a more detailed integrated view of diet and the CHD mechanism as well as its biomarkers were attempted.

The integrative view highlights the increased potential CHD risk that is associated with HGL diets. This potential risk is clearly elucidated in the wide range of CHD pathogenetic pathways which are mediated by HGL diets and the large array of CHD biomarkers which are affected as vividly shown in the simplified “connection graph”. HGL diets do not only influence the lipid and metabolic biomarkers, but also coagulation and vascular function biomarkers.

The use of α-glucosidase inhibitors is also found as substantially beneficial in CHD prevention efforts in diabetic patients by controlling important pathways shown in the integrated view of CHD. This further emphasises the importance of blood glucose and insulin levels in the prevention of CHD in diabetic patients. The array of biomarkers affected by these pharmacotherapeutic interventions would also indicate that these conditions could be of importance to non-diabetic patients.
